# Sensitization of TRPA1 by Protein Kinase A

**DOI:** 10.1371/journal.pone.0170097

**Published:** 2017-01-11

**Authors:** Jannis E. Meents, Michael J. M. Fischer, Peter A. McNaughton

**Affiliations:** 1 Department of Pharmacology, University of Cambridge, Cambridge, United Kingdom; 2 Institute of Physiology, Uniklinik RWTH Aachen, Aachen, Germany; 3 Institute of Physiology and Pathophysiology, University of Erlangen-Nuremberg, Erlangen, Germany; 4 Center for Physiology and Pharmacology, Medical University Wien, Wien, Austria; 5 Wolfson Centre for Age-Related Diseases, King’s College London, London, United Kingdom; Indiana University School of Medicine, UNITED STATES

## Abstract

The TRPA1 ion channel is expressed in nociceptive (pain-sensitive) somatosensory neurons and is activated by a wide variety of chemical irritants, such as acrolein in smoke or isothiocyanates in mustard. Here, we investigate the enhancement of TRPA1 function caused by inflammatory mediators, which is thought to be important in lung conditions such as asthma and COPD. Protein kinase A is an important kinase acting downstream of inflammatory mediators to cause sensitization of TRPA1. By using site-directed mutagenesis, patch-clamp electrophysiology and calcium imaging we identify four amino acid residues, S86, S317, S428, and S972, as the principal targets of PKA-mediated phosphorylation and sensitization of TRPA1.

## Introduction

The Transient Receptor Potential Ankyrin 1 (TRPA1) channel is expressed in nociceptive (pain-sensitive) somatosensory neurons, and activation of the channel triggers a sensation of stinging pain [1–4]. TRPA1 responds to a wide range of chemically diverse agonists, including natural products such as allyl-isothiocyanate (AITC, present in mustard oil and wasabi) and carvacrol (present in oregano) [2,5,6]; environmental toxins, such as acrolein (present in cigarette smoke) and nicotine [7,8]; common pharmaceuticals, such as lidocaine and propofol [9,10]; as well as a range of endogenous mediators [11,12] and protons [13]. TRPA1 has also been proposed to be activated by noxious cold [4,14,15] but this remains controversial [2,16]. Both intracellular and extracellular calcium can activate TRPA1 and potentiate its response to several agonists, followed by long lasting inactivation of the channel [2,16–18]. In contrast, under calcium-free conditions TRPA1 is strongly sensitized by prolonged application of agonists, through a slow shift of the channel’s voltage-dependence to more negative membrane voltages, and it has been proposed that this agonist-induced sensitization is important for the pain caused by prolonged exposure to irritants and allergens which activate TRPA1 [19].

Because many of its agonists can be inhaled, TRPA1 has become an important target for the treatment of inflammatory airway diseases [20] (reviewed in [21]). Upon tissue inflammation, immune and other cells release a range of inflammatory mediators, which modulate ion channels and thereby sensitize the responses of peripheral nociceptors. Over the past decades, we and other labs have characterized many such interactions, down to the individual phosphorylation sites (reviewed in [22]) and we have recently shown that inhibiting the interaction of kinases with the TRPV1 ion channel can be effective in reducing inflammatory pain [23,24].

TRPA1 is sensitized by bradykinin or by activators of proteinase-activated receptor (PAR) 2 via activation of intracellular pathways involving protein kinase A (PKA) and phospholipase C (PLC) [25,26]. Both pathways have been shown to induce increased membrane expression of TRPA1 [27]. However, a more recent study failed to show PKA-mediated sensitization of TRPA1 in DRG neurons using patch-clamp electrophysiology [28]. Nerve growth factor (NGF) is another important inflammatory mediator that increases TRPA1 expression [1,14,29]. Moreover, acute application of NGF was found to cause a sensitization of AITC-induced responses in certain DRG neurons [30].

Here, we investigate the acute sensitization of human TRPA1 caused by phosphorylation by PKA. We confirm that activation of PKA by forskolin (FSK) causes a strong sensitization of TRPA1-mediated responses. By using site-directed mutagenesis, we identify four serine residues critical for PKA-mediated sensitization of TRPA1.

## Materials and Methods

### Plasmids, mutagenesis and cell culture

The mTRPA1 and rat TrkA plasmids in pcDNA3.1 expression vectors were obtained from Dr Xuming Zhang (University of Aberdeen, Scotland). The hTRPA1 plasmid in a pTRE2 tetracycline-inducible expression vector was obtained from Dr Carla Nau (University Medical Center Schleswig-Holstein, Lübeck, Germany). Point mutations from serine or threonine to alanine of the putative PKA phosphorylation sites were performed on hTRPA1 in a pcDNA5/FRT-IRES-YFP vector [19] using the QuikChange II XL site-directed mutagenesis (SDM) kit from Agilent Technologies (La Jolla, CA), following the manufacturer’s instructions. Primers contained non-overlapping sequences at their 3’ end to minimize primer-dimerization as described by Liu and Naismith [31]. All constructs were confirmed by sequencing (Department of Biochemistry, University of Cambridge, UK).

Potential PKA phosphorylation sites on hTRPA1 were sought using GPS2.1.1 [32], NetPhosK [33], ScanSite 3 [34], pkaPS [35], and Kinasephos 2.0 [36]. Potential phosphorylation sites were targeted when they were identified with high probability by one or more of these packages.

HEK293t cells were transfected using Metafectene (Biontex Laboratories GmbH, Martinsried/Planegg, Germany) according to the manufacturer’s instructions. Electrophysiological recordings or calcium imaging were performed on the day following transfection. All experiments were performed at room temperature.

### Calcium imaging

Ratiometric calcium imaging was performed using fura-2 as described [24]. HEPES-buffered extracellular solution was used for all experiments (in mM: 140 NaCl, 4 KCl, 1.8 CaCl_2_, 1 MgCl_2_, 10 HEPES, and 5 D-glucose, adjusted to pH 7.4). Cells were continuously superfused with extracellular solution or test solutions through a common outlet. The TRPA1 agonist carvacrol was applied six times (50 μM; 20 s; 4 min gap). For every cell, the change in fluorescence ratio after application of carvacrol was calculated between the baseline (mean fluorescence during 20 s immediately before application of carvacrol) and peak fluorescence ratio reached within 40 s after carvacrol application. Responses of each individual cell were visually controlled and accepted only if responses 4 to 6 had a clear rising and falling phase and if peak fluorescence ratio was at least 5% above baseline. In experiments to measure the sensitizing effects of PKA activation, the adenylate cyclase activator forskolin (FSK, 10 μM; 120 s) was applied after the fourth carvacrol application, which was chosen in order to avoid distortion by calcium-mediated desensitization which occurs after the first TRPA1 activation (see Results) [18]. The sensitization ratio was then calculated as response 6/response 4. We chose the second response after FSK application to allow a full development of sensitization. Response amplitudes are variable in some cells, so only cells where the ratio of the last two responses preceding FSK application was 0.5 < response 4/response 3 < 1.5 were considered for analysis (see S4 Fig). The average response 4/response 3 ratios of all analyzed cells was 0.98 ± 0.01 for WT control, 0.98 ± 0.02 for WT + FSK, 0.94 ± 0.02 for S86A, 1.0 ± 0.02 for S317A, 1.02 ± 0.03 for S428A and 1.04 ± 0.02 for S972A. Experiments were performed in at least two independent transfections using a minimum of four recordings per condition.

### Patch-clamp electrophysiology

Whole-cell voltage clamp was performed as previously described [19]. All recordings were performed in calcium-free HEPES-buffered extracellular solution (in mM): 140 NaCl, 4 KCl, 1 MgCl_2_, 10 HEPES, and 5 glucose, adjusted to pH 7.4. The internal solution contained the following (in mM): 140 KCl, 1.6 MgCl_2_, 2.5 MgATP, 0.5 NaGTP, 2 EGTA, and 10 HEPES, adjusted to pH 7.3. All experiments were performed at a holding potential of -60 mV. Cells were continuously superfused with extracellular solution or test solutions through a common outlet. Carvacrol (100 μM) was initially applied for 150 s to activate agonist-induced sensitization and to reach stable current levels [19] and then switched to repeated 15 s applications with 30 s gap (see Results). PKA was activated by application of FSK (10 μM) twice after carvacrol response 8 and 9. The average current amplitude of responses 10–12 was normalized to the average of pre-FSK responses 7 and 8. To analyse the effect of the PKA inhibitor H89 on TRPA1 current, the average of responses 7 and 8 (before FSK but during H89) was normalized to the average of responses 5 and 6 (before H89). This means that only two H89-affected responses could be analysed instead of four as was done for FSK and control.

### Chemicals

Carvacrol, AITC, phorbol 12-myristate 13-acetate (PMA), forskolin (FSK) and ionomycin were all purchased from Sigma-Aldrich (Gillingham, UK). Fura-2 AM was purchased from Invitrogen (Paisley, UK), H89 from Cayman Chemical (Ann Arbor, USA), and NGF from Promega (Southampton, UK). Stock solutions of Fura-2, AITC, PMA, FSK, H89 and ionomycin were prepared in DMSO. The working concentration of DMSO did not exceed 0.1%. NGF was dissolved in PBS. Carvacrol was dissolved in HEPES-buffered extracellular solution.

### Data analysis

All data are presented as mean ± SEM. To reject outliers that might be due to changes in the recording conditions, such as deterioration of the seal or changes in access resistance, sensitization values outside of mean ± 3*SD were ignored. Two groups of data containing ≥ 10 samples were compared using the unpaired Student’s t-test. Multiple groups were compared by one-way ANOVA and either Dunnett’s multiple comparison test or Bonferroni *post-hoc* analysis. Statistical analysis was performed using Prism 5 or 6 (GraphPad Software, Inc., La Jolla, USA). Significance levels are as follows: n.s., not significant; *p < 0.05; **p < 0.01; ***p < 0.001.

## Results

### hTRPA1 is acutely sensitized by activation of PKA

**Fig 1:**
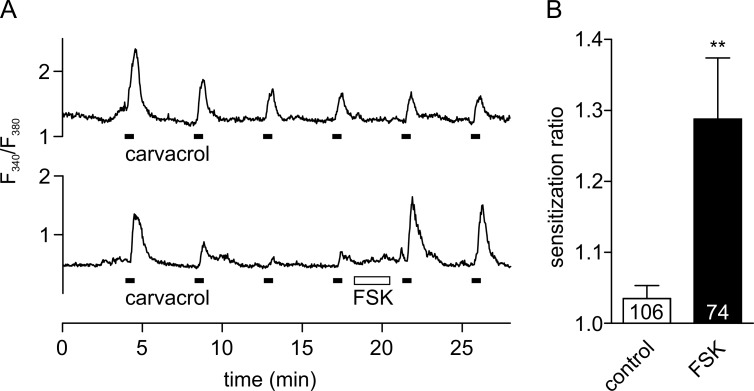
Activation of PKA by FSK sensitizes TRPA1. **A** Calcium-imaging experiments on hTRPA1-transfected HEK293t cells. TRPA1 was activated with six applications of carvacrol (filled bars; 50 μM; 20 s). Top: control experiment without application of FSK. Bottom: activation of PKA by application of FSK (open bar, 10 μM, 120 s) causes strong sensitization. **B** To allow the full development of sensitization, the sensitization ratio was calculated as the amplitude of the sixth over the fourth response. Sensitization ratio 1.04 ± 0.02 in control (white) and 1.29 ± 0.09 with FSK (black; *p* = 0.002, one-way ANOVA with Bonferroni post-hoc analysis). Number of cells under each condition is indicated. ** p < 0.01

Responses of TRPA1 to the covalent agonist AITC have been shown to be sensitized by activation of PKA [26]. Here, we confirm this role of PKA in calcium-imaging experiments on HEK293t cells, transfected with hTRPA1, using the non-covalent TRPA1 agonist carvacrol. Pulses of carvacrol evoked transient increases of [Ca^2+^]_i_ (Fig 1A). Calcium responses showed clear desensitization during the first two applications of carvacrol, a phenomenon that is well-known for TRPA1 and which is mediated by calcium entry [3,15,18]. However, responses stabilized after the first two carvacrol applications, allowing application of FSK to activate PKA after the fourth stimulus (Fig 1A). Responses following activation of PKA were significantly larger than in control recordings where no FSK had been applied (Fig 1A, bottom). In contrast, in pilot experiments using similar protocols neither NGF nor activation of protein kinase C (PKC) was found to acutely sensitize TRPA1 (S1 and S2 Figs).

**Fig 2:**
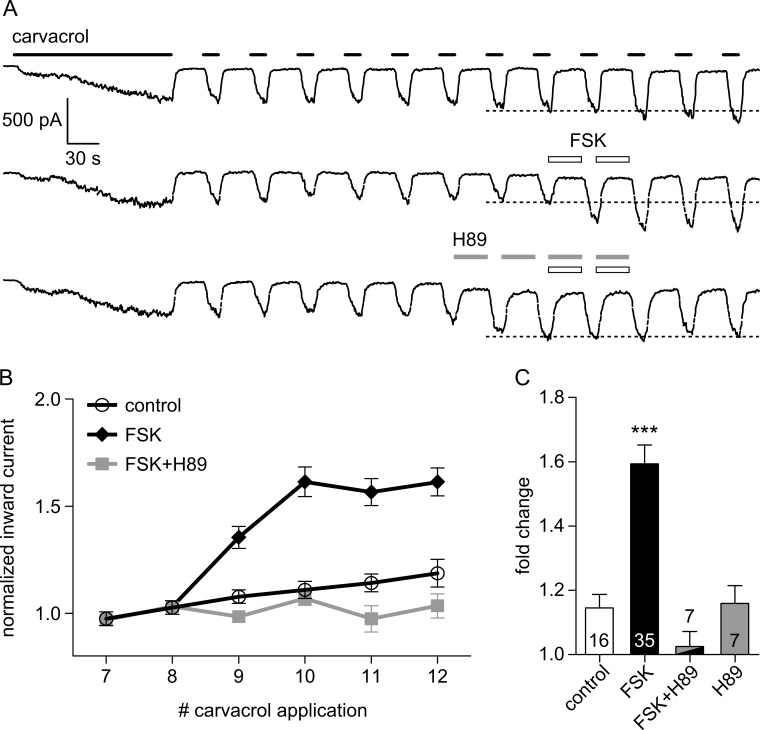
Sensitization of TRPA1 by FSK is PKA-dependent. Whole-cell patch-clamp recordings on hTRPA1-transfected HEK293t cells in calcium-free solution. **A** Carvacrol (100 μM) applied for 150 s before switching to repeated 15 s applications (30 s gap) produces a stable response pattern and avoids distortion by agonist-induced sensitization [19]. Calibration bar is the same for all traces. Dotted line shows average of pre-FSK responses 7 and 8. Top: control recording without application of FSK. Middle: Activation of PKA by FSK (10 μM; 2 x 30 s) sensitizes the following responses. Bottom: Application of the PKA inhibitor H89 (10 μM; 4 x 30 s) as indicated blocks sensitization by FSK. **B** Maximum inward currents at carvacrol applications 7–12 normalized to the average of pre-FSK responses 7 and 8 (dotted line in A). FSK (filled diamonds) causes a marked increase in current amplitude of responses 9–12 compared to control (open circles) and this effect is blocked by H89 (grey squares). **C** Quantification of B. Control: increase in mean of responses 10–12 was 1.15 ± 0.04; FSK: increase 1.59 ± 0.06 fold (*p* < 0.001 compared to control); FSK + H89: 1.03 ± 0.05 fold (*p* < 0.001 compared to FSK, *p* = 0.99 compared to control); H89: 1.16 ± 0.06 fold (*p* = 0.002 compared to FSK, *p* = 0.99 compared to control or compared to FSK + H89; all one-way ANOVA with Bonferroni post-hoc analysis). Total number of cells measured under each condition is indicated. *** p < 0.001.

These results were confirmed in whole-cell patch-clamp recordings (Fig 2). Calcium can both activate and desensitize TRPA1, therefore all patch-clamp recordings were performed in a calcium-free environment. We have previously shown that repeated application of agonists sensitizes hTRPA1, and that a prolonged pre-exposure to carvacrol achieves stable TRPA1 responses (Fig 2A) [19]. Application of FSK caused a significant increase of the carvacrol-induced inward current and this effect was completely abolished by the PKA inhibitor H89 (Fig 2C).

### Point mutations reveal four interaction sites for PKA on hTRPA1

**Fig 3:**
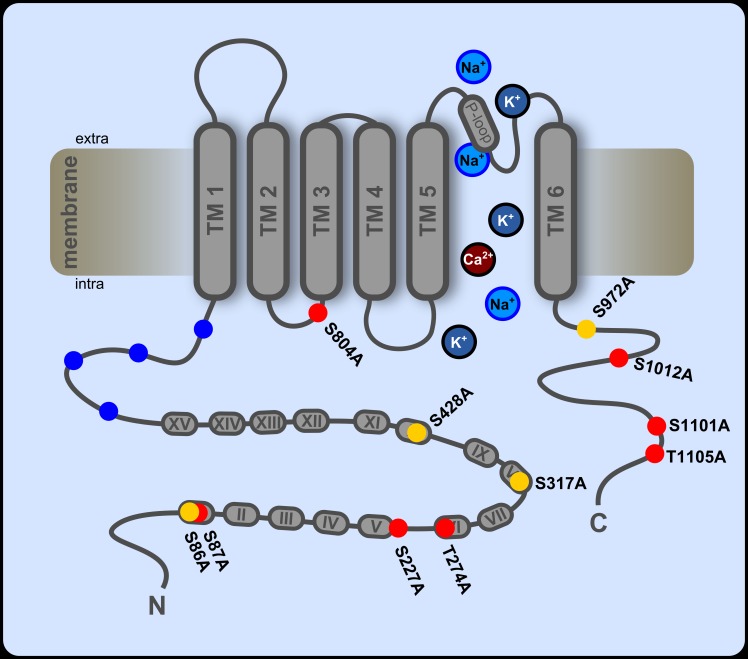
Location of the mutated residues on hTRPA1. Schematic representation of a TRPA1 monomer, indicating the location of the 11 putative PKA phosphorylation sites that were mutated to alanine. Red circles indicate mutations that did not reduce sensitization. Residues that were found to mediate sensitization by PKA are shown in yellow. Grey ovals indicate ankyrin repeat domains, numbered with roman numerals. Single cysteine and lysine residues (C621, C641, C665, K710) that have been found to be involved in channel activation by covalent agonists [38] are represented by blue circles. All positions are approximations based on the hTRPA1 sequence (NP_015628.2).

In order to characterize PKA-mediated sensitization in more detail, we set out to identify PKA phosphorylation sites on hTRPA1. Most likely candidate sites were screened using a range of web-based prediction tools (see Methods). Eleven serine or threonine residues were selected as having the highest predicted probabilities and were mutated one by one to alanine to disrupt phosphorylation by PKA, while affecting the functional environment of the channel as little as possible [37] (see Fig 3). Six mutations are N-terminal and are located within or in close proximity to ankyrin repeat domains. Four mutations are located in the C-terminal domain and one is in the linker between transmembrane domains two and three.

**Fig 4:**
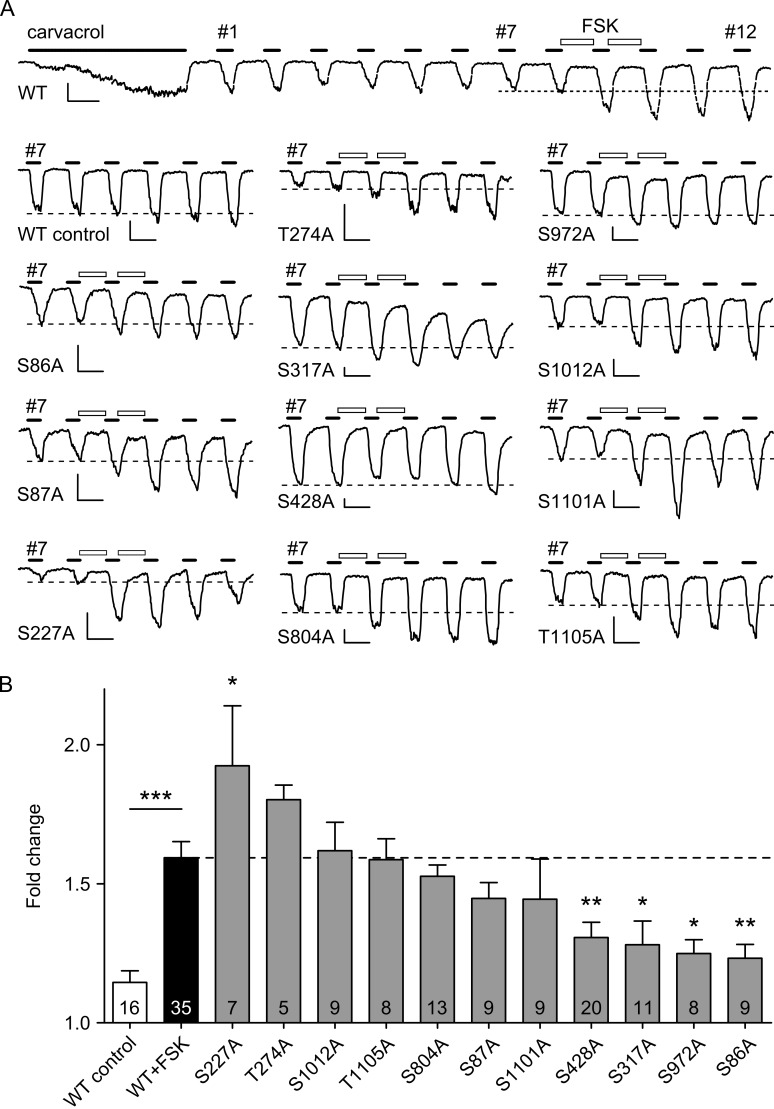
FSK-induced sensitization of TRPA1 S/T mutants in patch-clamp recordings. **A** Example traces showing sensitization of WT and mutant hTRPA1 by FSK (10 μM; 2 x 30 s). Full trace shown for WT hTRPA1; for mutant TRPA1, only carvacrol responses 7–12 are displayed. Response numbers are indicated. Dotted line shows average of responses 7 and 8. Scale bars in each trace are 200 pA and 30 s. Traces for WT and WT control have been replicated from Fig 2A. **B** Quantification of A. Values for WT control and WT+FSK are the same as in Fig 2C. In the WT control group (white), responses 10–12 were 1.15 ± 0.04 fold of responses 7 and 8. The sensitization by FSK (black) in the hTRPA1 WT is marked by the broken line (WT+FSK; 1.59 ± 0.06; *p* < 0.001). The following levels were measured in the hTRPA1 mutants (grey): 1.92 ± 0.22 for S227A (*p* = 0.048), 1.80 ± 0.05 for T274A, 1.62 ± 0.10 for S1012A, 1.59 ± 0.08 for T1105A, 1.53 ± 0.04 for S804A, 1.45 ± 0.06 for S87A, 1.45 ± 0.14 for S1101A, (all *p* ≥ 0.6), 1.31 ± 0.06 for S428A (*p* = 0.005), 1.28 ± 0.09 for S317A (*p* = 0.017), 1.25 ± 0.05 for S972A (*p* = 0.023), 1.23 ± 0.05 for S86A (*p* = 0.009). All one-way ANOVA with Dunnett’s test, compared to WT+FSK. The number of cells recorded in each group is indicated. * p < 0.05, ** p < 0.01, *** p < 0.001.

The hTRPA1 mutants were tested for FSK-induced sensitization in patch-clamp experiments. All mutants exhibited responses to carvacrol (Fig 4A) and did not influence TRPA1 current density, with the exception of the S1101A mutation, which displayed significantly increased current density (S3 Fig). In addition, none of the mutations affected TRPA1 activation kinetics, apart from the S804A mutation, which showed accelerated gating (S3 Fig). The majority of mutations displayed robust sensitization induced by FSK, indicating that these residues are not involved in sensitization by PKA. However, four mutations were found to display significantly reduced, albeit not completely abolished sensitization: S86A, S317A, S428A, and S972A (Fig 4B).

**Fig 5:**
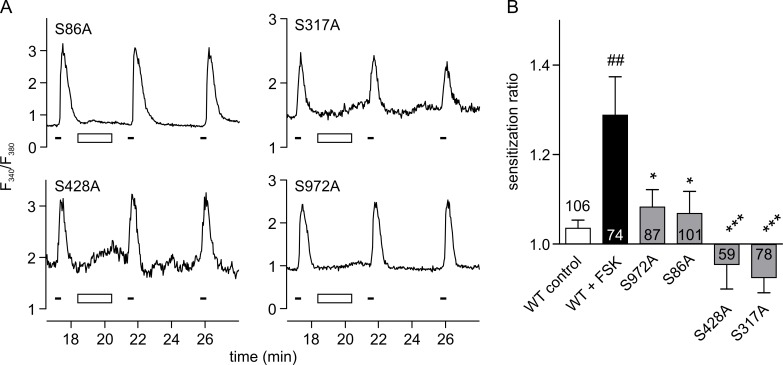
Sensitization of TRPA1 mutants in calcium imaging experiments. **A** Representative recordings from HEK293t cells expressing the four TRPA1 mutants which showed significant reduction in PKA sensitization in patch-clamp experiments (see Fig 4). The protocol was identical to that shown in Fig 1A. Only carvacrol responses 4–6 are displayed. **B** Quantification of A. Values for WT control and WT+FSK have been replicated from Fig 1B. In control experiments (white), sensitization ratio was 1.04 ± 0.02. With application of FSK (black) sensitization ratio was increased to 1.29 ± 0.09 (*p* = 0.002). Sensitization ratios in the four mutants (grey) were 1.08 ± 0.04 for S972A (*p* = 0.047), 1.07 ± 0.05 for S86A (*p* = 0.017), 0.95 ± 0.05 for S428A (*p* = 0.0002), and 0.92 ± 0.03 for S317A (*p* < 0.0001). Number of cells measured in each group is indicated. One-way ANOVA with Dunnett’s test, compared to WT+FSK. The number of cells recorded in each group is indicated. # compares to WT control; * compares to WT+FSK. * p < 0.05, *** p < 0.001.

In order to confirm the relevance of the four putative phosphorylation sites in a more physiological environment, they were tested using calcium-imaging experiments in the presence of normal levels of extracellular calcium. All four mutations showed significantly reduced sensitization; S317A and S428A abolished sensitization completely, whereas S972A and S86A decreased sensitization to a lesser extent (Fig 5).

Taken together, these results suggest the existence of four functionally significant phosphorylation sites for PKA on hTRPA1, namely S86, S317, S428, and S972.

## Discussion

The gating of TRPV1 is rapidly and potently enhanced when PKA or PKC are activated by a variety of inflammatory mediators, and also when TrkA is activated by the binding of NGF (reviewed in [22]). In preliminary studies we examined the ability of each of these signalling pathways to potentiate TRPA1. In agreement with other authors we found little short-term potentiation of TRPA1 by either PKC or by the NGF/TrkA pathway (S1 and S2 Figs). Activation of PKA, on the other hand, has a strong potentiating effect on gating of TRPA1, and so we focussed on elucidating the mechanism of action of this kinase.

Repeated activation of hTRPA1 by the non-covalent agonist carvacrol induces calcium increases in calcium-imaging studies (Fig 1) and inward currents in whole-cell patch-clamp studies (Fig 2). Application of FSK increased these responses both in calcium-free (patch-clamp) and calcium-containing solutions (calcium imaging) and this effect of FSK can be abolished by inhibition of PKA (Fig 2). This sensitizing effect of PKA on TRPA1 has been shown previously [26,27] but the sites of TRPA1 phosphorylation by PKA remained unknown.

We mutated 11 potential PKA phosphorylation sites in hTRPA1, identified using algorithms predicting likely phosphorylation sites, from serine/threonine to alanine. All 11 mutants were tested for effects on activation by agonist and sensitization by activation of PKA, in calcium-free patch-clamp recordings and using calcium imaging. One mutation, S1101A displayed increased current density and one other mutation, S804A, had some effect in speeding the time to peak in response to agonist. In the case of the S804A mutant, it is possible that its close proximity to the channel pore makes it relevant for gating kinetics. One mutant, S227A, may actually increase PKA-mediated sensitization, which seems counter-intuitive as the removal of a serine should have eliminated a potential phosphorylation site for PKA. A possible explanation may be that a conformational change induced by the amino acid substitution causes other PKA binding sites becoming more accessible. Four mutations, namely at positions S86, S317, S428, and S972, gave significant suppression of sensitization (Fig 4), suggesting that these are the functionally significant phosphorylation sites for PKA on hTRPA1.

Robust sensitization by PKA was observed in patch-clamp recordings under calcium-free conditions (Fig 2), but the sensitization seen in calcium-imaging experiments, with physiological levels of extracellular calcium, is considerably smaller (Fig 1). This may be explained by the strong desensitization of TRPA1 by calcium ions, the extent of which is well documented [3,15,18]; it has been proposed that an influx of extracellular calcium through an activated TRPA1 ion channel transfers the channel into an inactivated state that lasts for up to 20 min [18]. While such a long and complete inactivation was not seen here (4 min gaps between applications were enough for the channel to become fully responsive again), the possibility that a long-lasting desensitization by calcium interferes with the FSK-induced sensitization must be considered.

The calcium-imaging results show that mutation of either S317 or S428 is sufficient to completely disrupt PKA-mediated sensitization of TRPA1. The same residues had also been identified in calcium-free patch-clamp recordings. Two additional mutations, S86A and S972A had been identified as being involved in sensitization in calcium-free patch-clamp experiments but showed a lesser, though still significant, effect in calcium imaging studies. It is possible that S86 and S972 play a minor role in mediating sensitization by PKA, and that in a calcium-containing environment it is sufficient to ablate either of S317 or S428 in order to completely abolish sensitization by PKA. Another possibility is that, due to the decreased efficacy of sensitization in a calcium-containing environment, multiple phosphorylations need to occur to sensitize TRPA1 in a physiologically relevant manner.

**Fig 6:**
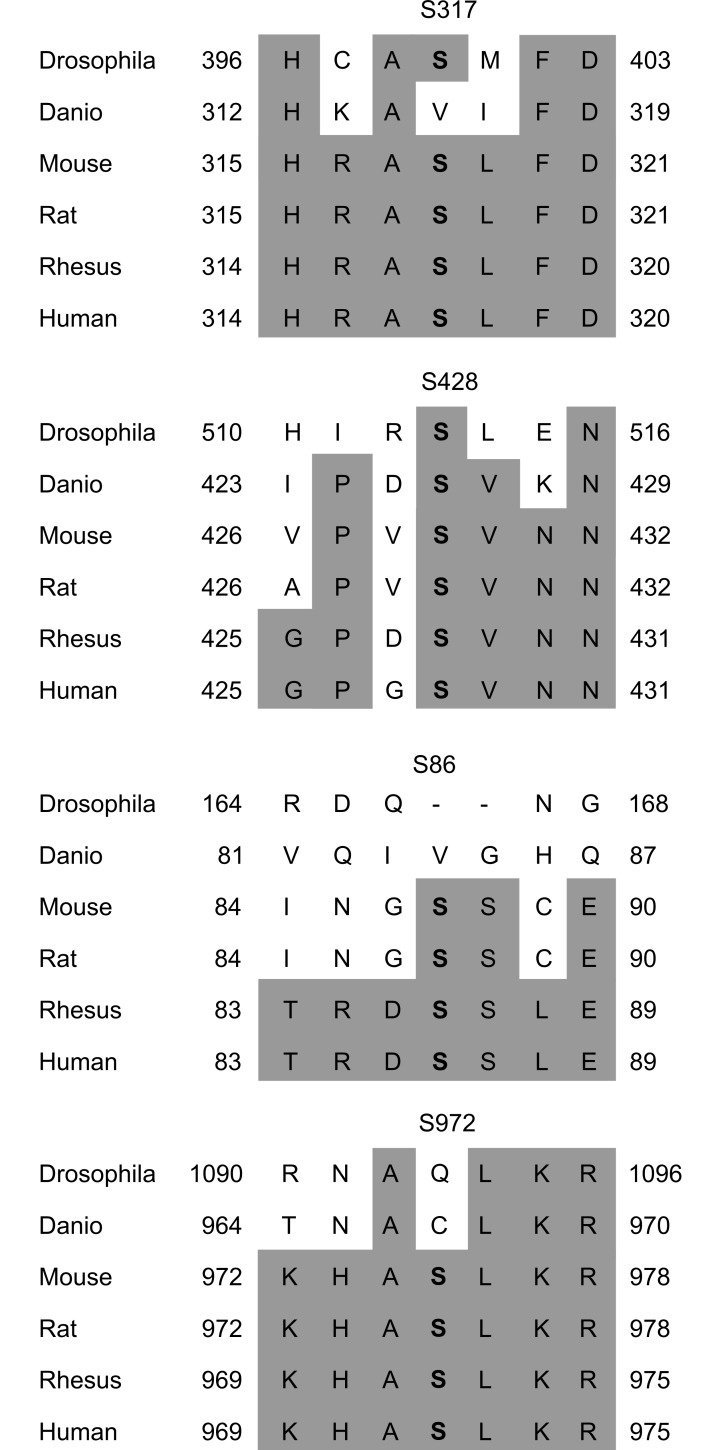
Alignment of TRPA1 protein sequence surrounding the four identified PKA phosphorylation sites. Sequence alignment carried out using Clustal Omega from EMBL-EBI (http://www.ebi.ac.uk/Tools/msa/clustalo/). Sequences for TRPA1 from Drosophila melanogaster (GenBank: AY302598.1; Viswanath et al., 2003), Danio rerio (zebrafish; GenBank: AAV37177.1), Mus musculus (mouse; Ref: NP_808449.1), Rattus norvegicus (rat; Ref: NP_997491.1), Macaca mulatta (rhesus macaque; Ref: XP_001083172.1) and human (Ref: NP_015628.2) obtained from NCBI. Shaded background indicates amino acids identical to the human isoform. Alignment is presented for S317 (top), S428 (second), S86 (third) and S972 (bottom). The serine residues of interest are marked in bold font. This alignment reveals that S317 and S428 are highly conserved across mammalian and non-mammalian species, whereas S86 and S972 are conserved across the mammalian species but are not present in Drosophila or zebrafish.

A sequence alignment of the TRPA1 protein between Drosophila, zebrafish, mouse, rat, rhesus macaque and human (Fig 6) confirms that both S317 and S428 are highly conserved. S317 is present in all species except zebrafish and S428 is present in all species examined. The two remaining sites, S86 and S972 are conserved across the mammalian species but are not present in Drosophila or zebrafish. Interestingly, in the case of S317, S428 and S972, the amino acids surrounding the putative phosphorylation sites are also highly conserved, suggesting they might act as a consensus motif required for protein kinase binding. In the case of S86, the surrounding area is only conserved between human and rhesus macaque.

How does phosphorylation at the four serine sites that we have identified influence TRPA1 channel gating to sensitize the responses? Three of the four relevant sites are located in the N-terminal domain of the channel (Fig 3), which is well known to be highly relevant for channel function. The remaining site, S972, is situated in the C-terminal part in close proximity to transmembrane domain 6. The published high-resolution crystal structure of TRPA1 (PDB ID 3J9P) [39] shows that S972 with its surrounding consensus motif is partially hidden behind other residues but is still solvent-accessible. This means that kinase binding to this residue is certainly possible but might be impeded to some extent. This could be a further indication for a minor role of S972 for general TRPA1 sensitization. The close proximity of this residue to the channel pore and to the putative voltage sensor of TRPA1 (R975, K988 and K989) [40] could explain how phosphorylation could lead to sensitization: the induced conformational change could act directly on the channel pore and the addition of a highly polar phospho-serine to the adjacent voltage sensor could influence voltage gating of TRPA1.

Unfortunately, no high-resolution crystal structure exists to date of the N-terminal structure of TRPA1 that contains the other phosphorylation sites S86, S317 and S428. These are all located within the ankyrin repeat domains, which have been proposed to influence the open probability of TRPA1. According to the model by Zayats and colleagues [41], the ankyrin repeat structure functions as a molecular spring, transducing mechanical force to the channel pore. It is possible that phosphorylation at S86, S317 or S428 influences the rigidity of the whole ankyrin repeat structure, similar to what was described for calcium binding to the N-terminal EF-Hand [41]. That might lead to a change in the force that is transduced to the channel pore and could thus facilitate channel opening and lead to sensitization. In that context, it is interesting to note that S86 is the most distal of these three phosphorylation sites and might therefore have the smallest effect on the general rigidity of the entire ankyrin repeat structure. That could explain why mutating S86 to alanine has less effect on sensitization.

As we have shown here, sensitization by PKA increases TRPA1 activity by less than twofold. It should be noted that other TRP channels show a more substantial sensitization. For example, TRPV1 was sensitized about 3-fold by PKA activation [42] and up to 6-fold by PKC activation [24]. In the case of TRPA1 however, rapid sensitization by inflammatory mediators is not the only mechanism to induce increased channel function as many irritants may also induce increased membrane expression of TRPA1 [27] and enhanced channel activity [19]. Taken together, increased TRPA1 function as a result of irritant exposure and inflammation seems to be a more complex mechanism that results from a combination of inflammatory sensitization, agonist-induced sensitization and membrane trafficking.

In conclusion, we show that activation of PKA sensitizes hTRPA1, and that this interaction is mediated by phosphorylation of four amino acid residues: S86, S317, S428, and S972.

## Supporting Information

S1 Fig Acute stimulation with NGF does not sensitize TRPA1 responses to AITC. **A** Representative recordings from ratiometric calcium imaging experiments on HEK293t cells, transiently transfected with mTRPA1 and TrkA. These were pilot experiments using a different protocol and isoform compared to figures in the main text. TRPA1 was activated with three applications of AITC (30 μM; 40 s; 10 min gap). Ionomycin was applied at the end to induce a maximum calcium response. Top: each AITC application triggers a calcium response of similar amplitude. Middle: cell additionally treated with NGF (100 ng/ml; 8 min) displays no change in its response to AITC. Bottom: mock transfected cell, treated with both AITC and NGF does not show any responses to either stimulus. **B**. Within cells that showed a stable response to the first two AITC applications, a sensitization ratio was calculated (response 3/response 2). In control experiments (white), a ratio of 1.21 ± 0.11 was obtained. Application of NGF (black) did not cause sensitization (0.99 ± 0.07; *p* = 0.11, unpaired t-test). The number of cells recorded in each group is indicated. It should be noted that these were pilot experiments performed on mTRPA1 before species differences in TRPA1 were known and further investigation into the acute effect of NGF on hTRPA1 should be conducted.
(TIF)

S2 Fig PKA but not PKC sensitizes hTRPA1 in HEK293t cells. **A** Representative recordings from ratiometric calcium imaging experiments on hTRPA1-expressing HEK293t cells (pTRE2 vector), co-transfected with GFP. These were pilot experiments using a different protocol and isoform compared to figures in the main text. TRPA1 was activated with four applications of carvacrol (50 μM; 20 s; 8 min gap). Top: each carvacrol application induces a calcium increase of similar magnitude. Second from top: cell treated with a combination of the PKC activator PMA (1 μM) and the PKA activator FSK (10 μM) for 120 s before the fourth stimulus shows a sensitized subsequent response. Third from top: cell treated only with FSK displays similar sensitization. Bottom: PMA alone did not increase carvacrol-induced responses. **B** A pronounced sensitization of TRPA1 was observed after PKA stimulation by FSK. The sensitization ratio (response 4/response 3) for control (white) was 1.08 ± 0.09, for FSK+PMA (light grey) 1.54 ± 0.13 (*p* = 0.023), for FSK alone (black) 1.55 ± 0.2 (p = 0.035), and for PMA alone (dark grey) 1.13 ± 0.05 (*p* = 0.99; all one-way ANOVA with Dunnett’s test against control). Number of cells in each group is indicated. * p < 0.05.
(TIF)

S3 Fig Effect of individual mutations on TRPA1 current density and activation kinetics. **A** Most mutations do not affect TRPA1 current density, measured at control response 8. Only the S1101A mutation caused a significant increase in current density. The following values were obtained for the individual mutants: 30.2 ± 6.4 for WT control, 31.6 ± 5.0 for WT+FSK, 71.1 ± 14.0 for S86A, 30.2 ± 7.9 for S87A, 21.8 ± 9.8 for S227A, 7.4 ± 2.1 for T274A, 68.2 ± 15.4 for S317A, 68.8 ± 8.4 for S428A, 34.5 ± 7.6 for S804A, 36.8 ± 10.6 for S972A, 45.7 ± 13.9 for S1012A, 6.5 ± 1.3 for T1105A (all p ≥ 0.3) and 130.9 ± 32.6 for S1101A (p < 0.001). **B** TRPA1 activation kinetics were measured at control response 8 as the time point of half-maximal inward current (half-response time). Only the S804A mutation caused a significant acceleration of channel opening. The following values were obtained for the individual mutants: 4.3 ± 0.3 for WT control, 4.2 ± 0.2 for WT+FSK, 4.2 ± 0.4 for S86A, 3.9 ± 0.3 for S87A, 5.3 ± 0.6 for S227A, 3.7 ± 1.3 for T274A, 4.2 ± 0.3 for S317A, 3.1 ± 0.2 for S428A, 3.8 ± 0.3 for S972A, 4.6 ± 0.5 for S1012A, 3.9 ± 0.5 for S1101A, 4.0 ± 0.7 for T1105A (all p ≥ 0.2) and 2.8 ± 0.2 for S804A (p = 0.04) (all one-way ANOVA with Bonferroni post-hoc analysis).
(TIF)

S4 Fig Stability of control responses 3 and 4 in calcium imaging experiments. The histogram shows the distribution of ratios of response 4/response 3 in all measured cells. Cells outside the range of 0.5 to 1.5 (dotted0020030lines) were considered extreme outliers and removed from data analysis.
(TIF)

S1 File Excel sheet containing data that led to the figures in the manuscript. (XLSX)
