# Supplementary figures and images for: Sensitization of TRPA1 by Protein Kinase A
Source: PLoS One. 2017 Jan 11;12(1):e0170097. doi: 10.1371/journal.pone.0170097 (PMC5226813; doi:10.1371/journal.pone.0170097)

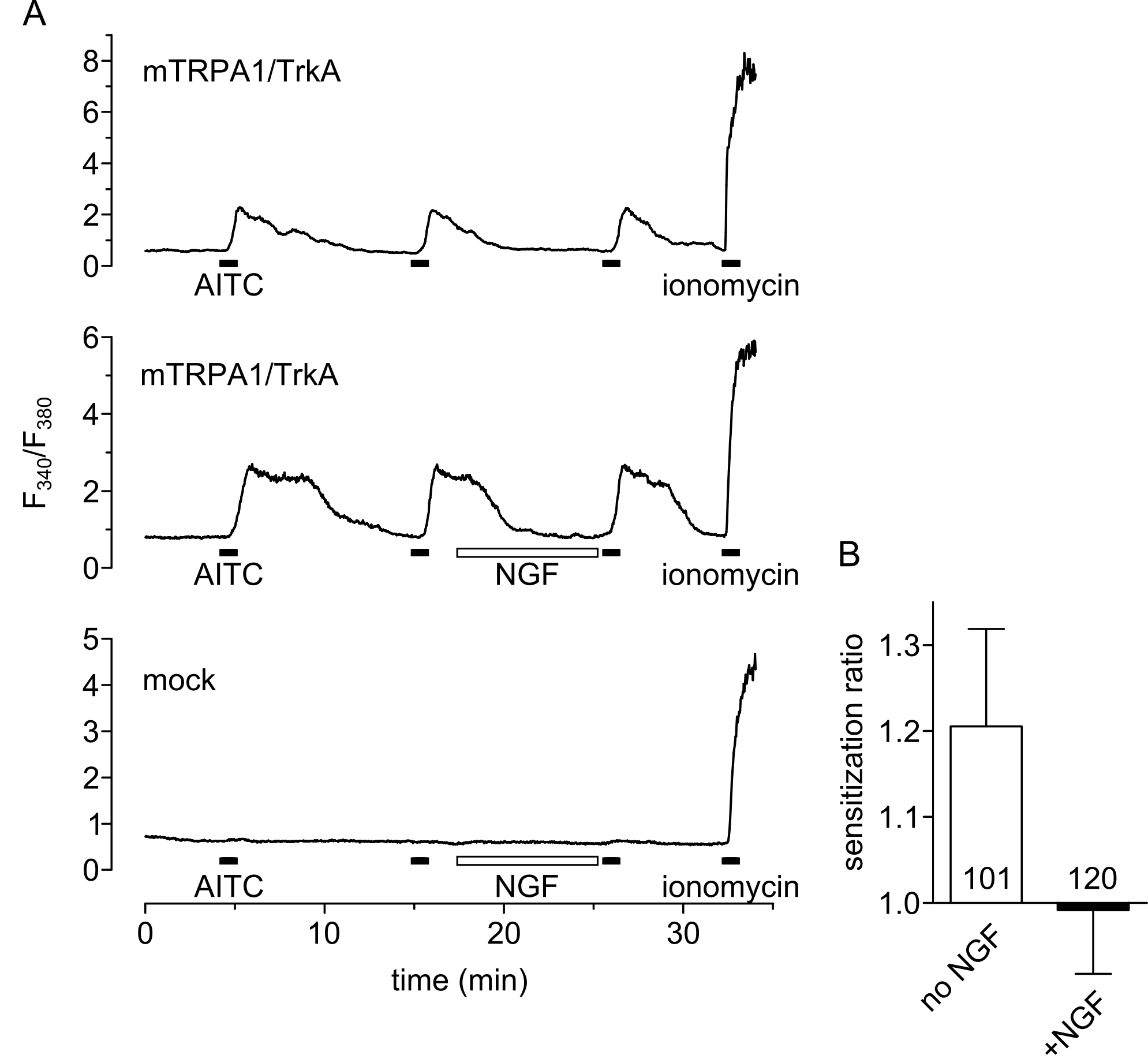

Supplement: S1 Fig — A Representative recordings from ratiometric calcium imaging experiments on HEK293t cells, transiently transfected with mTRPA1 and TrkA. These were pilot experiments using a different protocol and isoform compared to figures in the main text. TRPA1 was activated with three applications of AITC (30 μM; 40 s; 10 min gap). Ionomycin was applied at the end to induce a maximum calcium response. Top: each AITC application triggers a calcium response of similar amplitude. Middle: cell additionally treated with NGF (100 ng/ml; 8 min) displays no change in its response to AITC. Bottom: mock transfected cell, treated with both AITC and NGF does not show any responses to either stimulus. B. Within cells that showed a stable response to the first two AITC applications, a sensitization ratio was calculated (response 3/response 2). In control experiments (white), a ratio of 1.21 ± 0.11 was obtained. Application of NGF (black) did not cause sensitization (0.99 ± 0.07; p = 0.11, unpaired t-test). The number of cells recorded in each group is indicated. It should be noted that these were pilot experiments performed on mTRPA1 before species differences in TRPA1 were known and further investigation into the acute effect of NGF on hTRPA1 should be conducted. (TIF) [file pone.0170097.s001.tif]

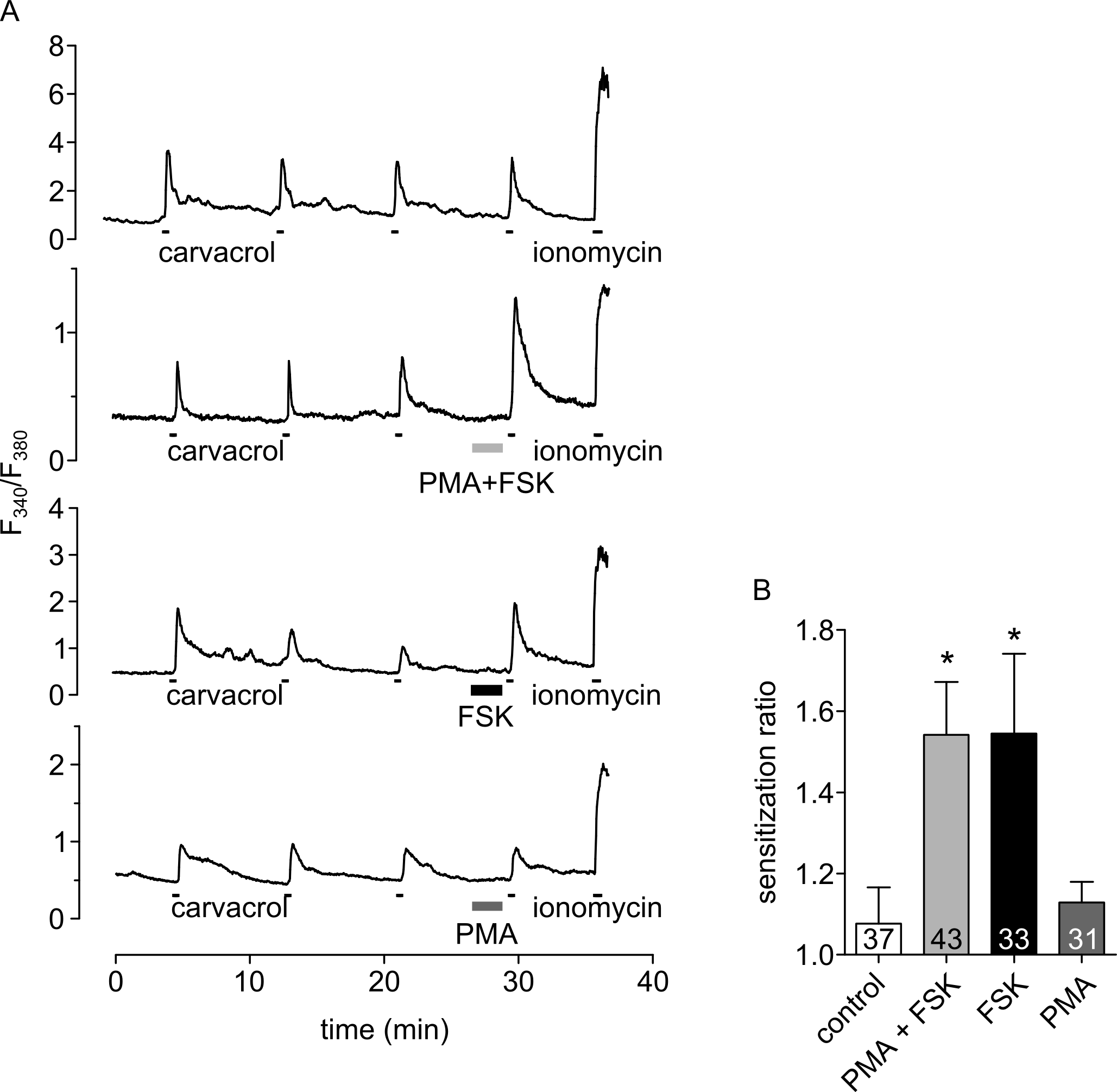

Supplement: S2 Fig — A Representative recordings from ratiometric calcium imaging experiments on hTRPA1-expressing HEK293t cells (pTRE2 vector), co-transfected with GFP. These were pilot experiments using a different protocol and isoform compared to figures in the main text. TRPA1 was activated with four applications of carvacrol (50 μM; 20 s; 8 min gap). Top: each carvacrol application induces a calcium increase of similar magnitude. Second from top: cell treated with a combination of the PKC activator PMA (1 μM) and the PKA activator FSK (10 μM) for 120 s before the fourth stimulus shows a sensitized subsequent response. Third from top: cell treated only with FSK displays similar sensitization. Bottom: PMA alone did not increase carvacrol-induced responses. B A pronounced sensitization of TRPA1 was observed after PKA stimulation by FSK. The sensitization ratio (response 4/response 3) for control (white) was 1.08 ± 0.09, for FSK+PMA (light grey) 1.54 ± 0.13 (p = 0.023), for FSK alone (black) 1.55 ± 0.2 (p = 0.035), and for PMA alone (dark grey) 1.13 ± 0.05 (p = 0.99; all one-way ANOVA with Dunnett’s test against control). Number of cells in each group is indicated. * p < 0.05. (TIF) [file pone.0170097.s002.tif]

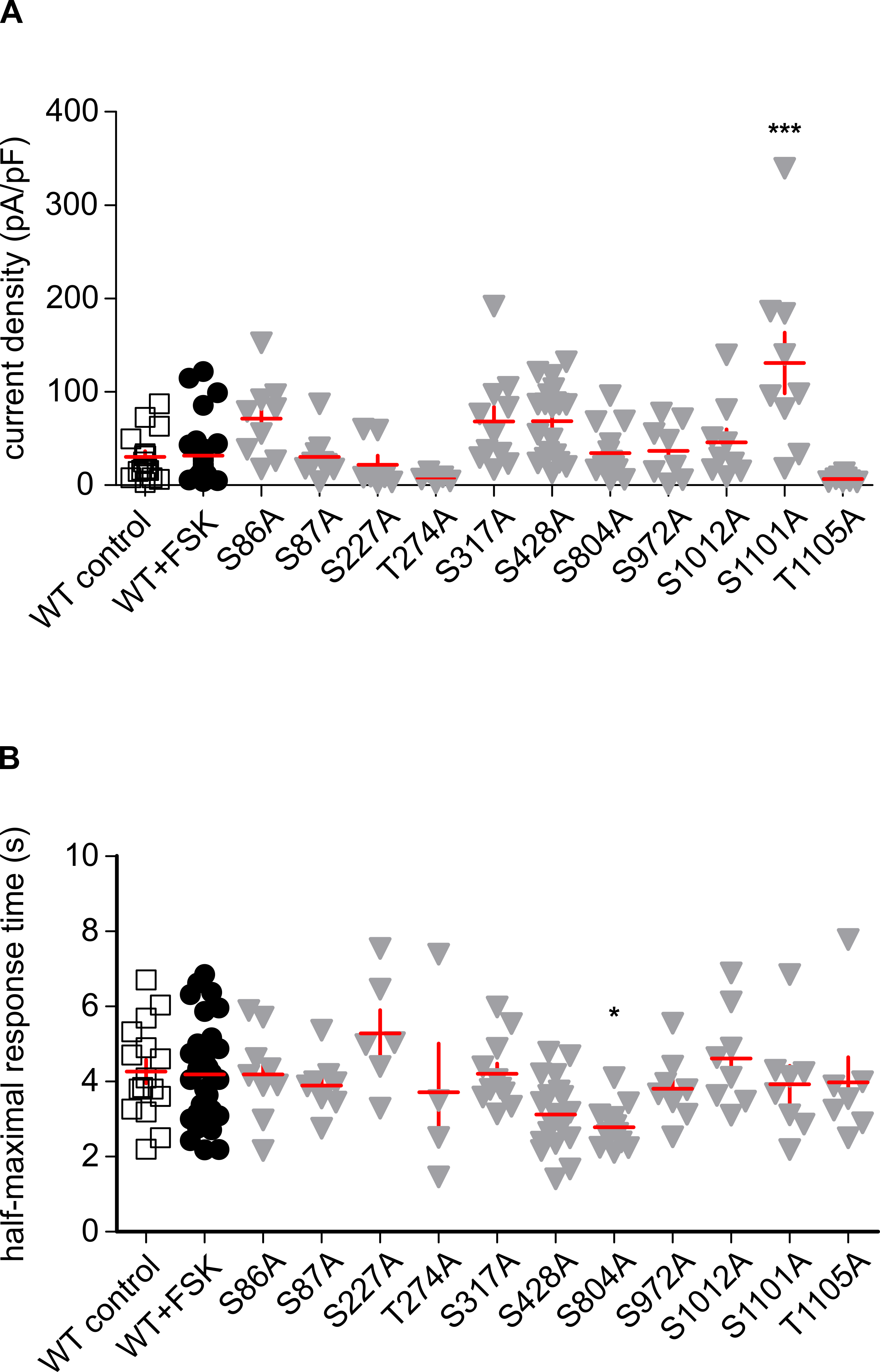

Supplement: S3 Fig — A Most mutations do not affect TRPA1 current density, measured at control response 8. Only the S1101A mutation caused a significant increase in current density. The following values were obtained for the individual mutants: 30.2 ± 6.4 for WT control, 31.6 ± 5.0 for WT+FSK, 71.1 ± 14.0 for S86A, 30.2 ± 7.9 for S87A, 21.8 ± 9.8 for S227A, 7.4 ± 2.1 for T274A, 68.2 ± 15.4 for S317A, 68.8 ± 8.4 for S428A, 34.5 ± 7.6 for S804A, 36.8 ± 10.6 for S972A, 45.7 ± 13.9 for S1012A, 6.5 ± 1.3 for T1105A (all p ≥ 0.3) and 130.9 ± 32.6 for S1101A (p < 0.001). B TRPA1 activation kinetics were measured at control response 8 as the time point of half-maximal inward current (half-response time). Only the S804A mutation caused a significant acceleration of channel opening. The following values were obtained for the individual mutants: 4.3 ± 0.3 for WT control, 4.2 ± 0.2 for WT+FSK, 4.2 ± 0.4 for S86A, 3.9 ± 0.3 for S87A, 5.3 ± 0.6 for S227A, 3.7 ± 1.3 for T274A, 4.2 ± 0.3 for S317A, 3.1 ± 0.2 for S428A, 3.8 ± 0.3 for S972A, 4.6 ± 0.5 for S1012A, 3.9 ± 0.5 for S1101A, 4.0 ± 0.7 for T1105A (all p ≥ 0.2) and 2.8 ± 0.2 for S804A (p = 0.04) (all one-way ANOVA with Bonferroni post-hoc analysis). (TIF) [file pone.0170097.s003.tif]

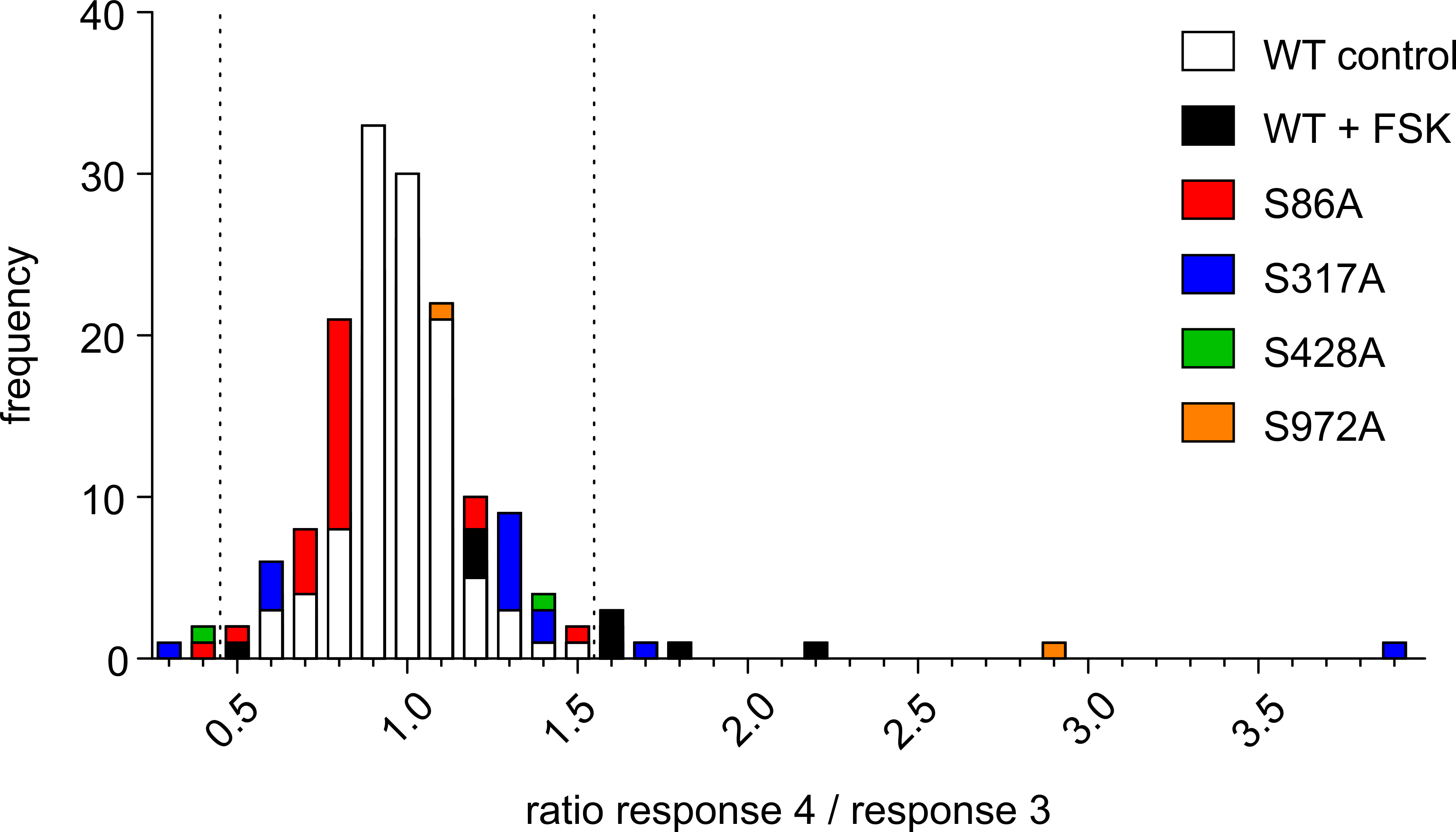

Supplement: S4 Fig — The histogram shows the distribution of ratios of response 4/response 3 in all measured cells. Cells outside the range of 0.5 to 1.5 (dotted0020030lines) were considered extreme outliers and removed from data analysis. (TIF) [file pone.0170097.s004.tif]
